# Twenty Years of Rad-Hard K14 SPAD in Space Projects

**DOI:** 10.3390/s150818178

**Published:** 2015-07-24

**Authors:** Vojtěch Michálek, Ivan Procházka, Josef Blažej

**Affiliations:** Dept. of Physical Electronics, Faculty of Nuclear Sciences and Physical Engineering, Czech Technical University in Prague, Břehová 7, 115 19 Prague 1, Czech Republic; E-Mail: blazej@fjfi.cvut.cz

**Keywords:** SPAD, rad-hard, photon counting, space

## Abstract

During last two decades, several photon counting detectors have been developed in our laboratory. One of the most promising detector coming from our group silicon K14 Single Photon Avalanche Diode (SPAD) is presented with its valuable features and space applications. Based on the control electronics, it can be operated in both gated and non-gated mode. Although it was designed for photon counting detection, it can be employed for multiphoton detection as well. With respect to control electronics employed, the timing jitter can be as low as 20 ps RMS. Detection efficiency is about 40 % in range of 500 nm to 800 nm. The detector including gating and quenching circuitry has outstanding timing stability. Due to its radiation resistivity, the diode withstands 100 krad gamma ray dose without parameters degradation. Single photon detectors based on K14 SPAD were used for planetary altimeter and atmospheric lidar in MARS92/96 and Mars Surveyor ’98 space projects, respectively. Recent space applications of K14 SPAD comprises LIDAR and mainly time transfer between ground stations and artificial satellites. These include Laser Time Transfer, Time Transfer by Laser Link, and European Laser Timing projects.

## 1. Introduction

One hundred years ago, Elster and Geitel imagined the first photon counting device [1]. It was based on *α* particles detector design introduced by Rutherford and Geiger. In a gas-filled tube with high voltage between electrodes, an electron is emitted by photon impact and accelerated by high electric potential causing gas ionization and detectable discharge. Such a device derived from Geiger–Müller counter was constructed by Locher in 1932 and was able to detect photons of visible light [2]. Another photon counting device which predecessor was invented at the beginning of 20th century is Photon Multiplier Tube (PMT) based on primary electron multiplication by secondary emission. Although PMTs are still manufactured today with very interesting parameters (high count rate, large sensitive area, practical absence of deadtime) they are not suitable for all applications, among others because of their low ruggedness, vacuum and high voltage necessity, and low sensitivity in red and near-infrared spectral regions [3]. The solid state detectors are much more rugged and are usually operated at much lower voltage with generally higher sensitivity. The most common solid state photon counting detector is the semiconductor Avalanche Photodiode (APD) or more precisely Single Photon Avalanche Diode (SPAD) when operated in Geiger mode.

The key feature of photon counting technique is the ability of detection of very weak optical signals even several orders of magnitude below noise. It outperforms even high sensitive analog detectors in applications with short measurement time window or with high accuracy a priori knowledge of time of arrival of the signal [3]. For this reason, it is employed in applications such as time-resolved Raman and fluorescence spectroscopy, chemical and biological luminescence analysis, quantum cryptography, or optical ranging and lidar [4]. The photon counting process is in principle efficient and highly stable analog to digital converter with high dynamic range and sensitivity. Devices based on photon counting, e.g., lidars, are generally of lower mass and power consumption compared to devices based on multiphoton approach, thus suitable for airborne and spaceborne applications. The main disadvantage of photon counting technique is the necessity of relatively (in compare with multiphoton approach) long integration time during which the conditions have to remain sufficiently unchanged. The practical calculation of the integration time is a deal between repetition rate, temporal resolution, and signal to noise ratio. This is due to the need of statistical treatment of many submeasurements resulting in the final measurement.

Solid state photosensitive detectors suitable for photon counting applications have been developed in our laboratory for more than two decades. According to the target application, detectors based on various semiconductor materials and their compounds such as Si, Ge, GeSi, GaAsP, GaP, and InGaAs were designed and tested. Inspired by Haitz [5], one of the most promising structure was developed, a silicon based Single Photon Avalanche Diode (SPAD) manufactured by K14 process [6]. It is a so-called thin SPAD with several unique features. Its radiation hardness predetermines it for space applications.

## 2. Detector Features

### 2.1. Structure and Principle of Operation

**Figure 1:**
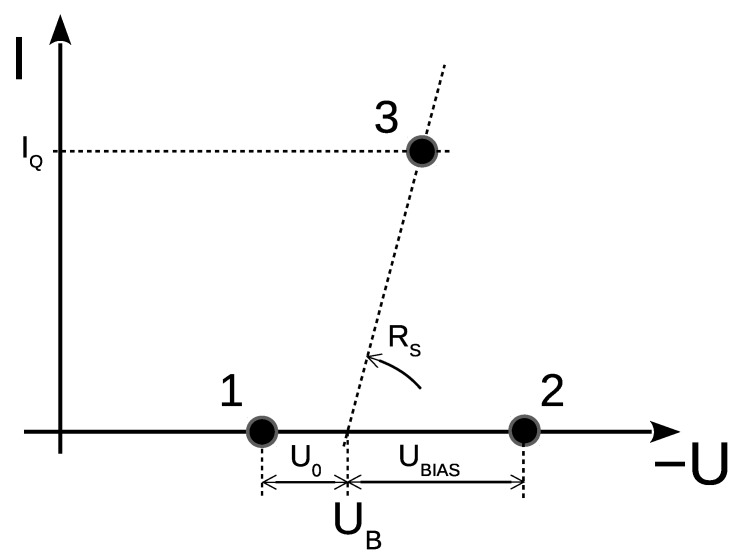
Current–voltage characteristic of a Single Photon Avalanche Diode (SPAD) operated in Geiger mode indicating three main states of the detection process. U_B_ denotes breakdown voltage.

A SPAD is typically operated in so called Geiger mode—indicating the analogy with gas filled detectors. The operation is based on reverse biased P-N junction of the SPAD above breakdown voltage. Standard operation cycle is depicted in I–V characteristic of the diode in Figure 1. At point 1, the bias is below breakdown voltage, thus the SPAD is disabled. Pushing the bias above breakdown voltage (point 2), the SPAD sensitivity is sharply increased. Due to an extremely high electric field inside the structure caused by a reverse bias on the thin p-n junction layer, the electron–hole pair generated by single incident photon or internal phonon is accelerated so that it generates another electron–hole pairs by collision with the semiconductor lattice. The new generated pairs collide again with the lattice, which results in self-sustaining avalanche—electric current multiplication (point 3). Based on employed circuitry, the avalanche is subsequently actively or passively quenched by decreasing the bias imposed on the P-N junction (point 1). At this moment, the diode can be again biased above breakdown voltage and next detection can occur [7]. The quenching time is an important property of the control electronics. For the active quenching nowadays reset times are limited by a transient delay of comparators and are in order of a few nanoseconds. For the passive quenching electronics the process is in principle stochastic and the actual value of reset time depends on a tolerance of application to the poorly defined detector state before next detection.

**Figure 2:**
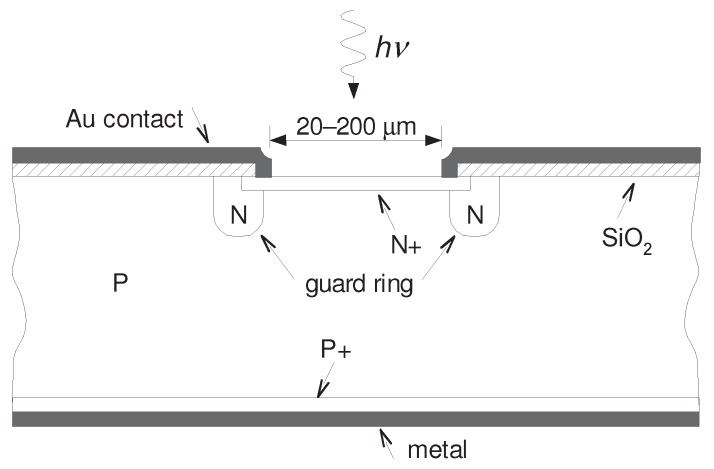
Cross-section of the K14 SPAD structure. The silicon bulk is p-doped, typical thickness of whole structure is 500 µm.

The SPAD sensor developed at Czech Technical University was manufactured by custom manufacturing process called K14, based on standard planar epitaxial technology [6]. The structure of K14 diode is depicted in Figure 2. The K14 diode is so-called thin SPAD pointing out that the depletion layer of p-n junction is thin, around 1 µm. Compared to thick SPADs (with depletion layer 20–150 µm), this results in better timing performance, low timing jitter and low breakdown voltage on order of tens of volts; about 28 V for K14. On the other hand, thin SPADs have reduced quantum efficiency due to lower volume where the photon can be absorbed [6,8].

**Figure 3:**
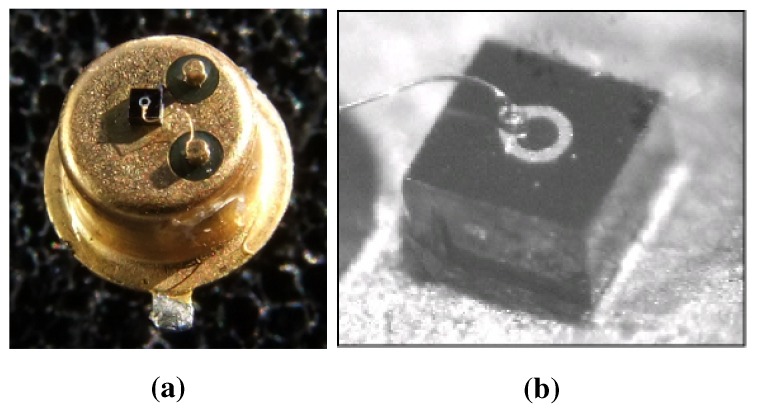
(**a**) The picture of K14 SPAD chip mounted onto a standard TO (Transistor Outline) package. The anode is at the bottom of the chip mounted to the package via an electrical conductive adhesive [9]; (**b**) The detail of the chip active area diameter of 100 µm with wire bonding of the diode cathode. The sensitive area is inside the circle formed by cathode metal contact [6].

There is a guard ring around the photon sensitive area made of n-type semiconductor reducing the danger of microplasma breakdown caused by steep gradient typical for SPAD sensors. Moreover, the guard ring restricts the multiplication area to specific region [3]. There were manufactured K14 diodes with sensitive area of several diameters ranging from 20 µm to 200 µm with outstanding uniformity regardless of the sensitive area. The pictures of a K14 SPAD chip can be found in Figure 3.

**Figure 4:**
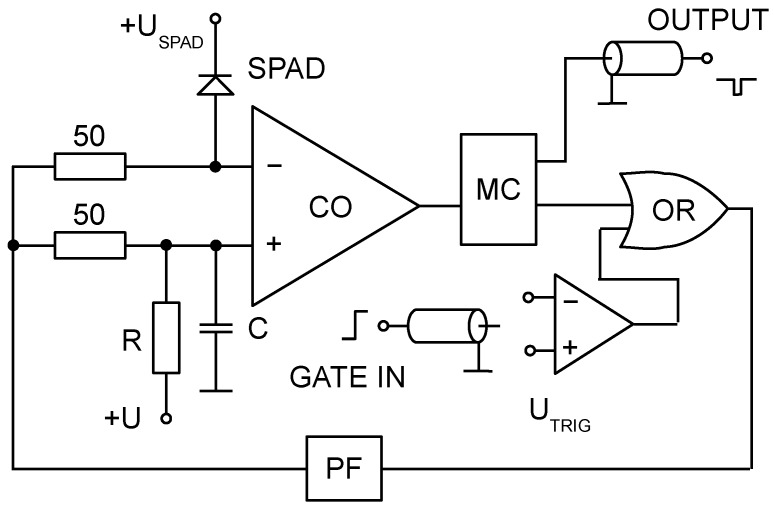
Block diagram of active quenching and gating circuit [10]. The reset (quenching) time of detector is determined mainly by a propagation delay of the comparator CO and the gate OR.

The K14 SPAD is operated coupled with a custom made active quenching and gating circuits inspired by Cova [11]. These slightly vary according to target application in which the detector containing the K14 diode is used. A common block diagram of such a circuitry can be found in Figure 4. The avalanche is sensed by a comparator (CO). The generated pulse is stretched by a monostable circuit and logically ORed with gate input. The output drives a pulse-forming circuit, output of which controls the bias on the SPAD.

Beside active quenching, also passive quenching may be employed. In such a circuit, there is a resistor in series with the diode. When a breakdown occurs, the current increase due to the avalanche causes increase of voltage at the resistor, hence, decrease of voltage at the diode, resulting in avalanche quenching. The advantage over active quenching is simplicity of the circuit. On the other hand, active quenching may be used at higher count rates [8]. Not only because the quenching time is generally longer in passive case, but also because the passive circuit results in longer biasing time of thediode [12].

### 2.2. Detector Properties

Several parameters of the K14 SPAD and its supporting electronics are described in following section. Especially parameters which are outstanding [4] compared to other SPAD detectors are emphasized. The point of view on each parameter depends on intended application [13]. The dark count noise of the K14 SPAD is relatively high, but for many space application with high background flux its importance is negligible. On the other hand, its stability of temporal response and uniformity of sensitivity within sensitive area allow few-photon recognition [14] and multi-photon compensation [15].

**Figure 5:**
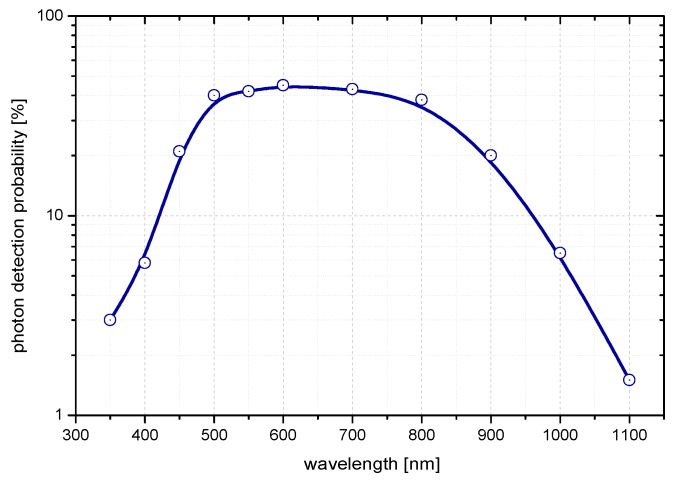
Typical detection probability versus wavelength [16] for a chip with a sensitive area of 20 µm and 1 V bias above breakdown voltage at room temperature.

The K14 sensor spectral sensitivity with 1 V bias voltage at room temperature is depicted in Figure 5 and is determined by its core material—silicon. Note that the detection probability is increased with increasing bias voltage and it is red-enhanced with increasing temperature of detector. It can be up to five times higher for 4 V bias above breakdown voltage. Selected bias voltage is always a deal between the detection probability, temporal resolution and dark count probability. All three factors are directly proportional to the bias voltage.

**Figure 6:**
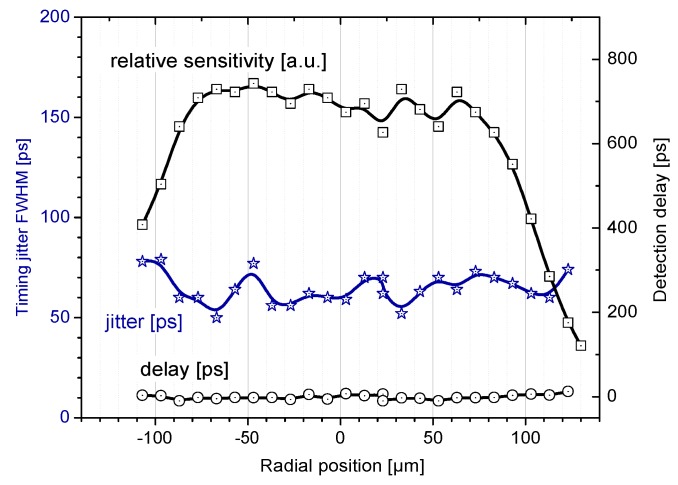
Uniformity of a typical 200 µm K14 SPAD. Note the detection delay, independent on position of seed photoelectron. This uniformity is absolutely crucial property for any photon-number dependent application such as few-photon detection and multi-photon time-walk compensation. It is also necessary for application where exact position of incident photon is unpredictable, e.g. in focus of a large telescope.

One of the unique properties of the K14 SPAD is its uniformity. That means the detection delay remains the same regardless on the position in active area, in which the incident photon is absorbed. This maintains high temporal resolution and low jitter even for detectors with large sensitive area (200 µm) [6]. An experiment was performed to verify the K14 uniformity [6]. A light spot of size of few micrometers scanned the surface of active area by employing micrometer shifts with accuracy of few micrometers. Relative sensitivity, timing resolution and detection delay across the SPAD structure was measured at several points of the active area. The results are depicted in Figure 6. Note that the data spread of the detection delay is caused by a statistical nature of the measured parameter.

**Figure 7:**
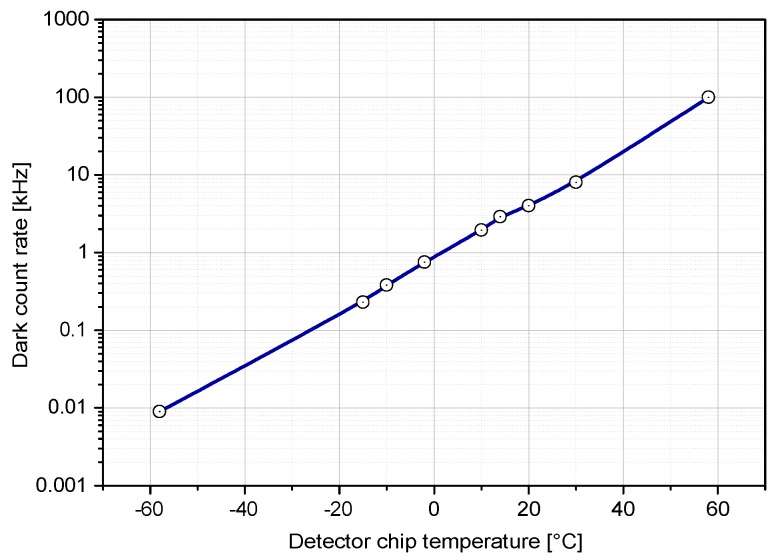
Dark count rate of the detector is strongly dependent on temperature of the structure. Depicted dependence is valid for typical K14 SPAD of 40 µm active area operated 0.7 V above breakdown voltage. Note the slope of one decade decrease of dark count rate per 30 K. The absolute value of dark count rate depends on bias, see Figure 8.

An electron–hole pair generation and avalanche establishment can occur after biasing of the SPAD even without the incident photon. This events are caused by lattice thermal vibrations together with crystal defects and impurities, and they are called dark counts. These cause false detections and determine the internal noise of the detector. The dark counts—thus the noise—can be diminished by cooling the sensor [17]. Dependence of dark count on temperature of K14 diode is shown in Figure 7.

**Figure 8:**
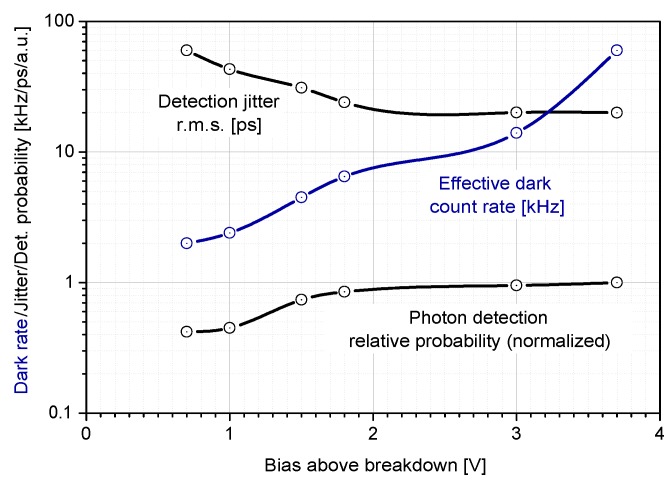
Three important and interconnected parameters of typical 200 µm K14 sensor cooled at −60 °C plotted against the bias voltage.

Most of the monitored properties of the K14 SPAD are dependent on the bias. Some properties (see below) are better with higher bias operation, some of them deteriorate. For this reason, the bias have to be carefully selected with respect to target application. Having in mind key detector properties for respective application, a trade-off is usually undertaken [18]. Three important detector parameters—timing jitter, dark count rate, and detection probability—are plotted against the bias in Figure 8. Note that with increasing bias, the detection probability and timing jitter is improved while the number of darkcounts increases.

**Figure 9:**
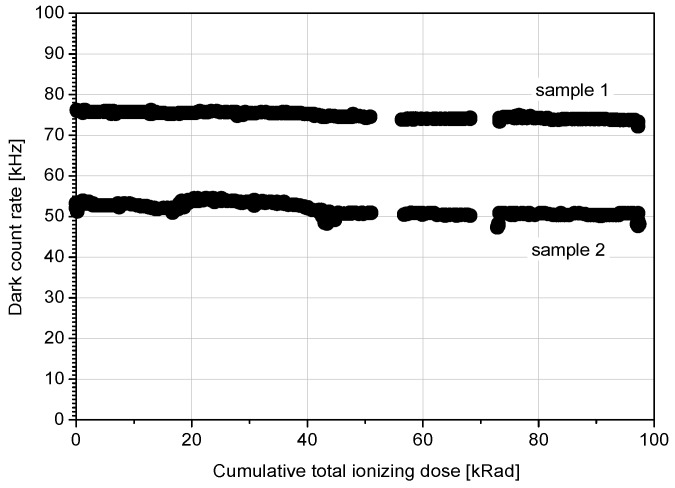
Dark count rate stability of two selected K14 100 µm diodes while irradiated. Note two different dark count rate values demonstrating variation in dark count rate sample by sample whereas it is independent on imposed radiation dose.

An important feature of the K14 sensor is its radiation hardness. This was verified during the years in three main tests. Moreover, the radiation hardness was proved in practice via in orbit operation onboard Jason-2 and COMPASS satellites. The terrestrial radiation tests were performed in three different laboratories. One of them were performed in CNES laboratory in Toulouse, France in frame of T2L2 project. The diodes underwent Total Ionizing Dose (TID) of 100 krad. The dark count rate of the detector operated in Geiger mode was recorded regularly as the dose grew, see Figure 9.

**Figure 10:**
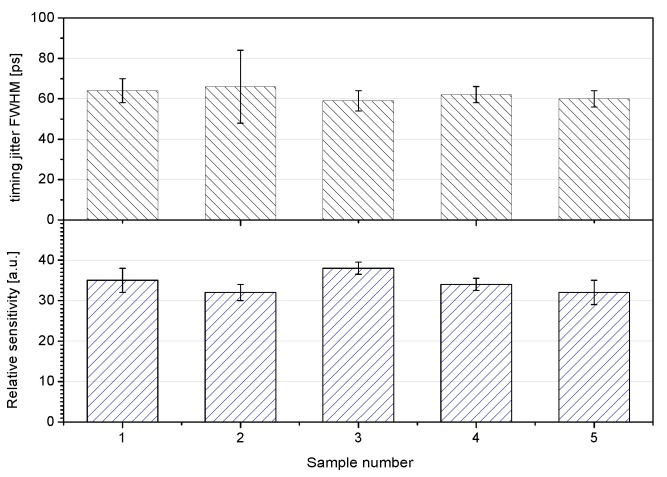
Timing resolution and sensitivity of reference (not irradiated samples) 1, 4, 5 and 100 krad irradiated samples 2, 3. The irradiated samples does not exhibit any measurable parameter change.

Beside the dark count, timing resolution and sensitivity were monitored after the irradiation. In Figure 10, these parameters are compared among irradiated and not irradiated samples of the diodes. The experiment has shown that the K14 SPAD is fairly radiation resistant and has no measurable change in monitored parameters—dark count rate, timing resolution, and sensitivity—after TID of 100 krad [18].

Next radiation experiment was performed at Nuclear Research Institute in Řež, Czech Republic. A radiation source of ^60^Co was employed in a geometrical setup that results in radiation speed of 2.5 rad/s. The total irradiation time was 225 min resulting in TID of 34 krad. Similarly to above mentioned experiment, such a radiation dose did not degrade monitored parameters—timing jitter, dark count rate, and breakdown voltage. However, it must be noted that the present gamma radiation increase the effective dark count rate during the experiment as the gamma photons triggered the avalanche. The dark count rate increased from 0.2 MHz to 1.2 MHz. It dropped back to 0.2 MHz after the source of gamma radiation was shielded [18].

Final radiation experiment with K14 SPADs were performed at Indiana University Cyclotron Facility in Bloomington, USA. In comparison with above mentioned experiments, a proton flux instead of gamma rays was employed as a radiation source. Various fluxes of 53 MeV protons struck the K14 diodes in several scenarios—diode operated in active quenching and gating circuit, diode under steady bias below breakdown voltage, and diode with shorted pins. In contrast to gamma radiation, proton flux caused significant deterioration (increase) of dark count rate due to displacement damage in the structure of the diode. This happened for much lower TID than used in previous gamma rays experiments. It was shown that the increase in dark count rate is not only dependent on TID but also on proton flux value. The higher value of proton flux, the higher increase in dark count rate was observed. The diodes exposed to proton fluxes of values in range of 10^4^ cm^−2^s^−1^ to 2.3 × 10^8^ cm^−2^s^−1^ exhibit increase of dark count rate on average from 0.27 MHz to 0.55 MHz at TID of 0.3 krad, 2.33 MHz at 3 krad, and 4.9 MHzat 6.65 krad [19].

**Figure 11:**
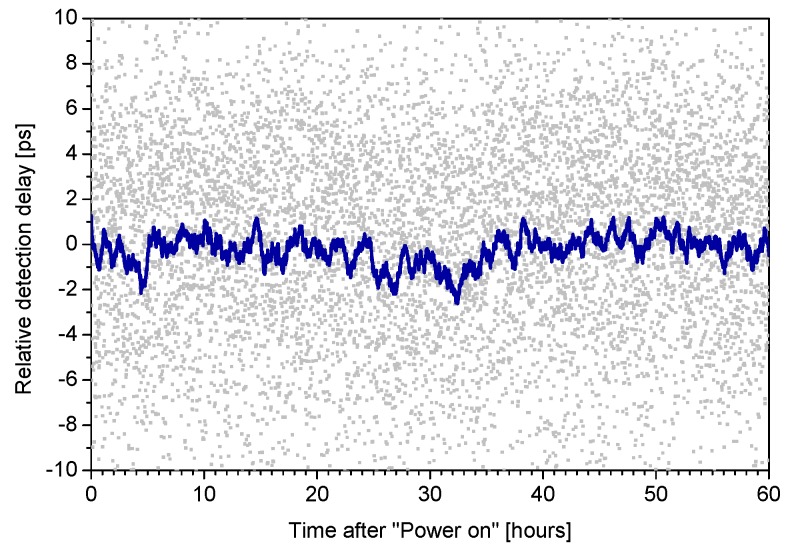
The verification of detector stability. Detection delay readings averaged over 1000 s during a Time-Correlated Single Photon Counting (TCSPC) experiment [20] in more than two days.

**Figure 12:**
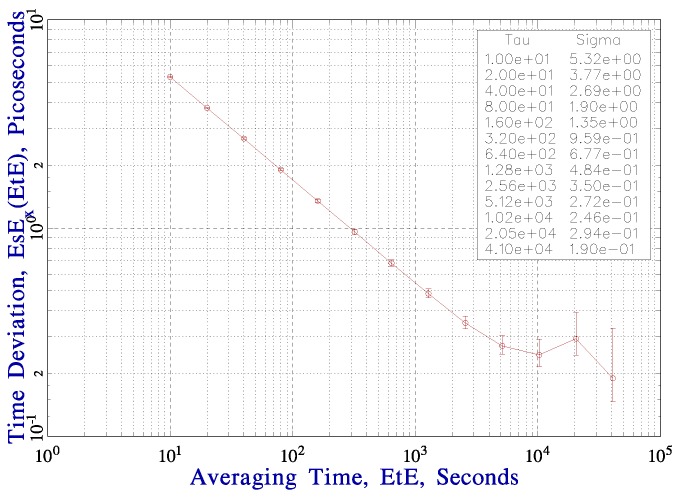
Time deviation of the entire TCSPC experiment [20]. In the sense of TDEV the long-term detection stability is better than 200 fs after several hours. In other words, it is meaningful to process data collected in several hours acquisition.

One of the most important key features of the K14 sensor and the detector electronics for intended applications is its detection delay stability. The stability was among others verified in a Time Correlated Single Photon Counting (TCSPC) experiment lasting more than 2 days. A short pulse (42 ps) laser diode of 778 nm wavelength and in house built NPET timing system (New Picosecond Event Timing device) with sub-picosecond resolution and great timing stability [21] was employed. The results are depicted in Figure 11 and Figure 12. Figure 11 shows delay detection measurements averaged over 1000 s. The delay remains within ±1 ps while the ambient temperature fluctuations during the experiment were within ±1 K. Figure 12 shows time deviation (TDEV) of the whole TCSPC experiment including stabilities of laser, start detector and NPET timing system. Note the plateau below 200 fs at averaging times of few hours. Same results were obtained for thermo-electrically cooled 200 µm chips as well as 100 µm chips without thermal management [20].

**Figure 13:**
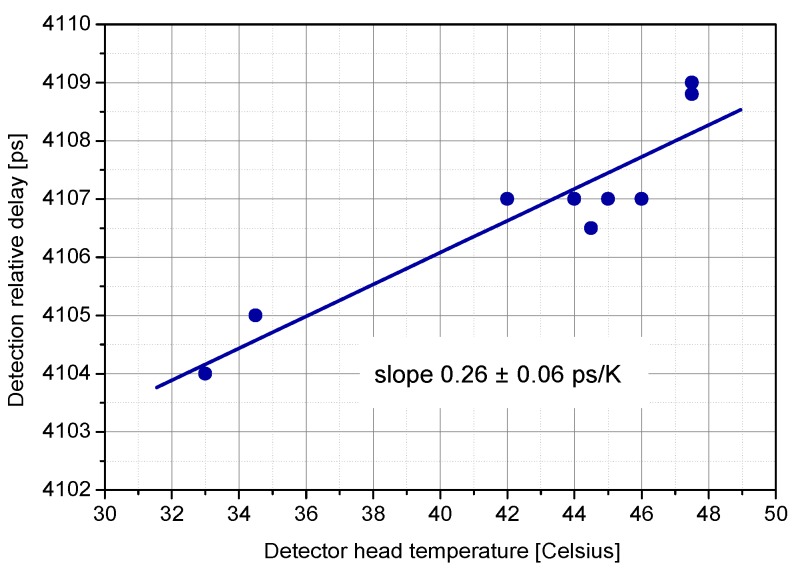
Temperature dependence of detection delay [20] for typical chip and active quenching circuit with the built-in temperature compensation.

The detection delay stability is affected by mainly two influences, namely, temperature and the number of incident photons. The dependence of detection delay on temperature changes was measured in a TCSPC experiment with the result of 0.26 ps/K, see Figure 13.

**Figure 14:**
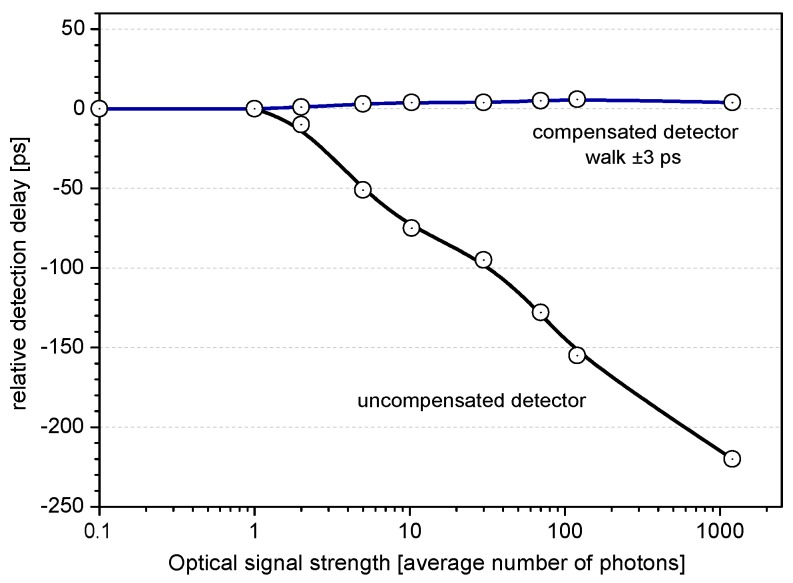
Detection delay of compensated (C-SPAD) [7] and uncompensated SPAD dependence on number of incident photons. The core of ground-segments of many photon-counting space projects.

Another detection delay fluctuations are caused by varying optical input strength, thus incident number of photons. The degree of such an influence is dependent among others on voltage bias and pulse width in case of multiphoton level. The level of so called time walk is not acceptable for sub-centimeter Satellite Laser Ranging (SLR). For this reason, an effort in compensation of such an effect was made. It was shown that the rise time of avalanche pulse is slightly shorter for multiphoton level than for single photon level. This observation was employed by the SLR group in Graz to develop a time walk compensating circuit [15]. The detection delay dependence on the number of incident photons of compensated and uncompensated K14 detector is shown in Figure 14. Another way to mitigate the time walk caused by varying signal strength is to ensure the single-photon level at the detector input; this approach is used for instance in the European Laser Timing project [22].

Note well the number of incident photons mentioned above means number of signal photons from the laser pulse concentrated in few tens of picosecond. On the other hand, the detection delay is fairly stable under various background photon flux in range of 1 to several million photons per second when operated in gated mode.

## 3. Space Projects

The K14 SPAD has been implemented in several space projects. In the mid-nineties, the SPAD was part of a single photon lidar device which was developed at the Space Research Institute in Moscow, Russia, for the MARS94 mission. The device was suitable for the mission for several reasons. First of all, low mass, dimensions and power consumption was advantageous for the aerostat carrier. Moreover, due to photon counting principle, not only altitude measurements but also atmospheric backscatter signal and consequently dust and aerosols densities may be obtained. The lidar was intended to measure altitude of the aerostat from 0 to 5 km above the Mars surface with resolution of 5 m while its altitude changes with respect to sun position, heating the balloon filled with CO_2_ and increasing the lift [23]. The balloon with density close to the density of atmosphere (gently lower during day, gently higher during night) allows passively and repetitively to transport scientific load in low atmospheric layers. Unfortunately, due to launch problems, the probe did not reach the correct space orbit and the mission was untimely canceled [7].

A similar, analogous device was used in late nineties for NASA mission Mars Surveyor ’98, the Mars Polar Lander spacecraft. The probe was to land in south polar region of the Mars. The lidar device was intended to contribute by atmospheric dust and haze monitoring to one of the mission goals—recording local meteorological conditions [24]. The lidar device was mounted on top of the lander with upward view and can work in either active or passive mode. The active lidar shots laser pulses and monitors the backscattered signal from particles in the atmosphere up to 750 m. In passive mode, the detector monitors the sun light scattered by atmospheric particles up to 100 km above the surface. The sun illuminates consecutive layers from upper to lower and vice versa during sun rise and sun set, respectively. In this way, the vertical profile of the atmosphere up to highest layers may be obtained [25]. Unfortunately, few minutes prior to the atmospheric entry, the communication with the probe was lost and was no more resumed [26].

**Figure 15:**
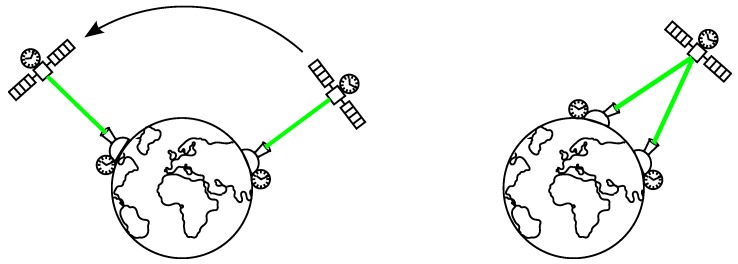
Diagram of optical time transfer via satellite comprising both common view mode (**right**) and non-common view mode (**left**).

Recent projects in which the K14 SPAD is involved concentrate on optical time transfer. The optical time transfer methods are by one order more accurate than methods employing microwaves as the propagation delay of the optical signal through the atmosphere is much precisely modeled than that for microwaves, mainly because of ionosphere contribution [27,28]. Optical time transfer via satellites allows synchronization of ground clock with satellite clock or even time transfer between ground stations clock on intercontinental distance. An example diagram of the time transfer via satellite can be found in Figure 15. The technique exploits existing Satellite Laser Ranging (SLR) stations. The SLR laser pulses fired towards the satellite are time-tagged using a ground station clocks. Then, the photons of laser pulse are detected by the satellite detector and time-tagged by the satellite clock. Finally, the photons reflected by retroreflector of the satellite are detected by the ground station and time-tagged again using SLR station clock. Using this triplet arrival times, mutual state of the satellite and ground station time scales can be obtained. In non-common view mode, the satellite can carry the time information from one station to another by synchronizing the time scales of the stations between each other. Compared to the common view mode, it can transfer the time on larger distances where common view is unreachable; however, with less accuracy as the time transfer is affected by mid- and long-term stability of the satellite clock, more precisely by the inaccuracy of the clock model. In case of common view mode, only short-term stability (<1 s) is of importance [29].

**Figure 16:**
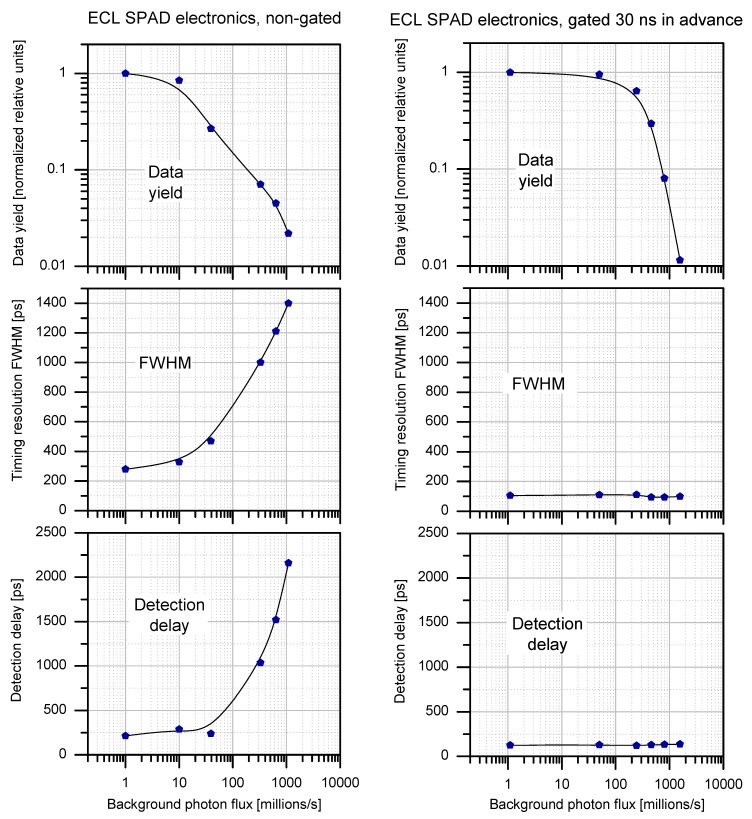
Comparison of gated and non-gated mode [10] of operation used in Laser Time Transfer (LTT) project.

The K14 SPADs are utilized in the French project Time Transfer by Laser Link (T2L2) [30]. In cooperation with CNES and CERGA, France (former part of Observatoire de la Côte d’Azur) high dynamic range SPAD detector was developed. It allows to detect photon counts from single to tens of thousands. The satellite detection device comprises two different optical detectors. One measures precisely the number of incident photons while the other determines their arrival time. The device is on-board the Jason-2 satellite orbiting in altitude of about 1300 km.

Another K14 SPAD resides on-board Chinese navigation satellites (BeiDou) on much higher orbit, altitude of 21,500 km [31]. The detectors were developed in cooperation with Chinese Academy of Sciences in frame of Laser Time Transfer project. In contrast to T2L2 detectors, these sensors operate merely in single photon mode. Although the detector possesses single-photon sensitivity, it withstands direct pointing to Sun without any damage. The first version of the detector [32] residing on COMPASS-M1 satellite operates in non-gated mode. The high background photon flux, however, limits the data yield and timing resolution. This leads to development of the detector allowing operation in both gated and non-gated modes. Comparison of gated and non-gated modes of operation is illustrated in Figure 16.

**Figure 17:**
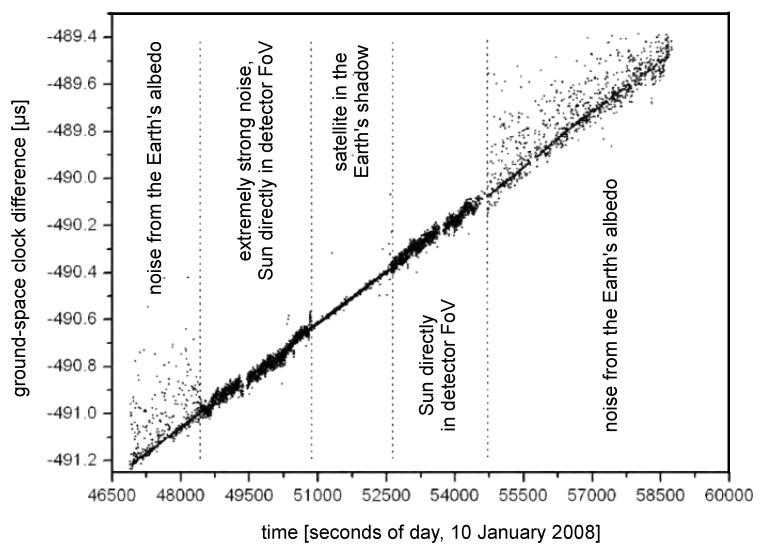
Comparison of ground hydrogen maser and on-board rubidium clock via optical time transfer in frame of LTT project. Several conditions are depicted [32]. This is typical configuration for M1 satellite, for other satellites the field of view has been modified to eliminate direct Sun exposure.

The results of the time transfer in various conditions may be seen in Figure 17. The satellite passes several attitudes with respect to Sun and Earth during the orbit. The background photons from direct sunlight or reflected from Earth surface strongly affects the time transfer performance. Typical precision of clocks time difference measurement is about 300 ps while the uncertainty of the measurement of the relative frequency difference is of about 3 × 10^−14^ in 2000 s [32].

**Figure 18:**
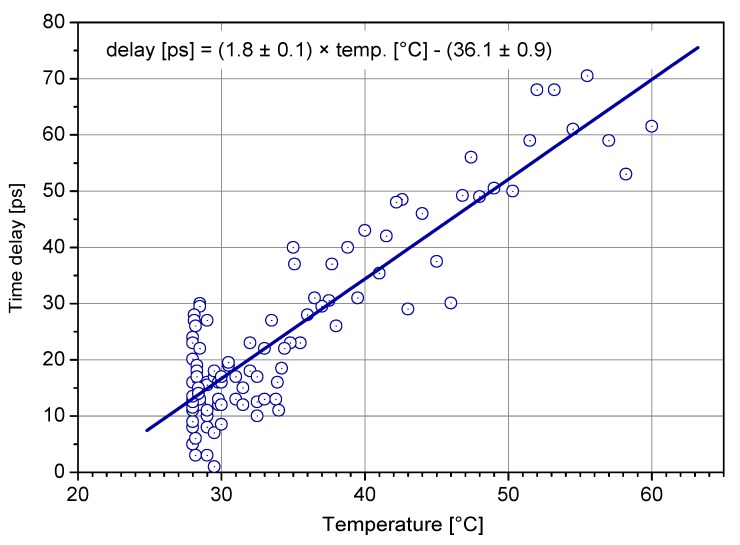
Detection delay against temperature of SPAD for European Laser Timing (ELT) project.

The most recent optical time transfer project comprising K14 SPAD is the European Laser Timing (ELT) [22]. It is part of an Atomic Clock Ensemble in Space (ACES) project which is to study the behavior of atomic clock in microgravity environment. The project includes the main microwave link for time transfer reaching precision of few hundreds of picoseconds. The optical time transfer part operating along with the microwave part allows to reduce the uncertainty down to approx. 25 ps and accuracy to about 100 ps [33]. The detector along with other electronics and optics shall reside on the International Space Station (ISS) 400 km above Earth surface and provide the synchronization of the ground and space clock. Considering the low Earth orbit and energies of SLR station lasers, the signal can reach multi-photon level at the input of detector in the space. Despite this, the photon counting approach was chosen in order to reduce the time transfer systematic errors as much as possible. For this reason, similarly to LTT project, single detector operating at single photon level is used; however, higher precision is required in compare with the LTT project. This is achieved by increasing the voltage bias, which, however, leads to increase in dark count rate, too. Fortunately, the effective dark count rate of un-cooled detector of several hundreds of kHz—which is rather high value—may be tolerated, as the background photon flux is roughly of the same order. The detection delay stability is again of high importance in order not to affect comparison of ground and space time scales. The the maximum variation of the delay at most 20 ps under the temperature fluctuations, within a single ISS revolution, is expected to be 6.5 K. The detection delay temperature dependency is plotted in Figure 18 reaching for the specified temperature range the detection delay change of approx. 11.4 ps, which safely meets the requirement. This result is achieved by employing special circuit compensating temperature variations of voltage bias, so that the bias above breakdown voltage is maintained at same value for whole range of operating temperatures [34]. Tough requirements are imposed on the absolute calibration of delays within the time transfer chain. The optical to electrical detection delay of the SPAD should be calibrated with uncertainty of at most 25 ps. For this purpose, new measurement technique had to bedeveloped [35].

Beginning at the SLR station in Graz, Austria, the compensated SPAD based on K14 has been operated at several SLR stations around the world [15]. As mentioned in the previous section, along with timing resolution, the SPAD delay stability independent on number of incident photons is of high importance in this application. The basic product of SLR stations is time of flight of a short laser pulse towards the satellite and back to station corrected for atmospheric delays. In this way, the distances between SLR stations and satellites are measured with sub-centimeter uncertainty. These data are important contribution to geodesy, geodynamics, and geophysics [36]. Beside the sub-centimeter accuracy satellite ephemerides, they provide data such as length of the day, SLR station coordinates and velocities, geocenter coordinates, coefficients of Earth gravity field, and several fundamental physical constants [36].

## 4. Conclusions

During the years, several valuable properties of silicon K14 sensor and its supporting electronics—such as resistance against gamma radiation or detection delay stability—has been utilized in various space projects. The detectors have been used in several projects utilizing (single) photon counting techniques. The K14 SPAD was a part of devices such as single photon planetary altimeter, atmospheric lidar or receiver for high accuracy laser time transfer. The detector is also embedded in the receiver part of several SLR stations around the world. Its future applications are expected in any mission utilizing single photon counting, e.g., optical time transfer missions or one-way optical navigation in the solar system.
